# Successful corticosteroid therapy for severe liver injury secondary to herbal traditional Chinese medicine, Mega Defends X, assessed for causality by the updated RUCAM: A case report

**DOI:** 10.1097/MD.0000000000039439

**Published:** 2024-08-23

**Authors:** Lu Chai, Ran Wang, Rolf Teschke, Shenghao Jin, Jiao Deng, Xingshun Qi

**Affiliations:** a Department of Gastroenterology, Liver Cirrhosis Study Group, General Hospital of Northern Theater Command (Teaching Hospital of Shenyang Pharmaceutical University), Shenyang, China; b Department of Internal Medicine II, Division of Gastroenterology and Hepatology, Klinikum Hanau, Hanau, Germany; c Academic Teaching Hospital of the Medical Faculty, Goethe University Frankfurt/Main, Frankfurt am Main, Germany; d School of Clinical Medicine, China Medical University, Shenyang, China; e Department of Pharmacology, General Hospital of Northern Theater Command (Teaching Hospital of Shenyang Pharmaceutical University), Shenyang, China.

**Keywords:** corticosteroid treatment, herb-induced liver injury, Mega Defends X, traditional Chinese medicine, Updated Roussel Uclaf Causality Assessment Method

## Abstract

**Rationale:**

In China, herbal traditional Chinese medicine products are readily obtained without any requirement of prescription and widely used in real life, thereby increasing the incidence of their related herb-induced liver injury (HILI). However, the diagnosis of HILI is still challenging because its clinical manifestations are variable due to lack of any specific biomarkers. Misdiagnosis and inappropriate treatment may result in the progression of HILI.

**Patient concerns:**

A 55-year-old female patient was admitted to the hospital due to progressive jaundice.

**Diagnoses:**

The diagnoses of HILI secondary to Mega Defends X, an herbal traditional Chinese medicine product, for which the score was 9 based on the updated Roussel Uclaf Causality Assessment Method of 2016.

**Interventions:**

The patient received corticosteroid with a stepwise dosage reduction.

**Outcomes:**

The liver injury significantly improved by corticosteroid treatment.

**Lessons:**

Corticosteroids should be potentially effective and safe in patients with severe HILI.

## 1. Introduction

As the key metabolic center of chemical ingredients found in herbal products, the liver is particularly susceptible to adverse toxic reactions including herb-induced liver injury (HILI).^[1]^ In a Chinese retrospective study on 1985 drug-induced liver injury (DILI) and HILI cases observed between 2009 and 2014, as much as 563 cases (28.4%) were HILI.^[2]^ Once HILI is diagnosed, suspected herbs should be withdrawn according to the current practice guidelines.^[3]^ Most of patients will recover spontaneously within 6 months of herb withdrawal. However, some patients will progress to chronic liver injury, acute liver failure (ALF), and even death.^[4]^ A meta-analysis found that 1.5% of the 936 cases with confirmed HILI developed chronic liver injury and 10.4% died.^[5]^

Prescription and non-prescription conventional drugs are common causes of DILI. As opposed, herbal products are causes of HILI with clear different features compared with DILI.^[6]^ We report the clinical course in a case of severe HILI secondary to Mega Defends X, an herbal product of traditional Chinese medicine, with focus on the effectiveness of corticosteroid therapy and causality assessing approach.

## 2. Case presentation

**Figure 1.**
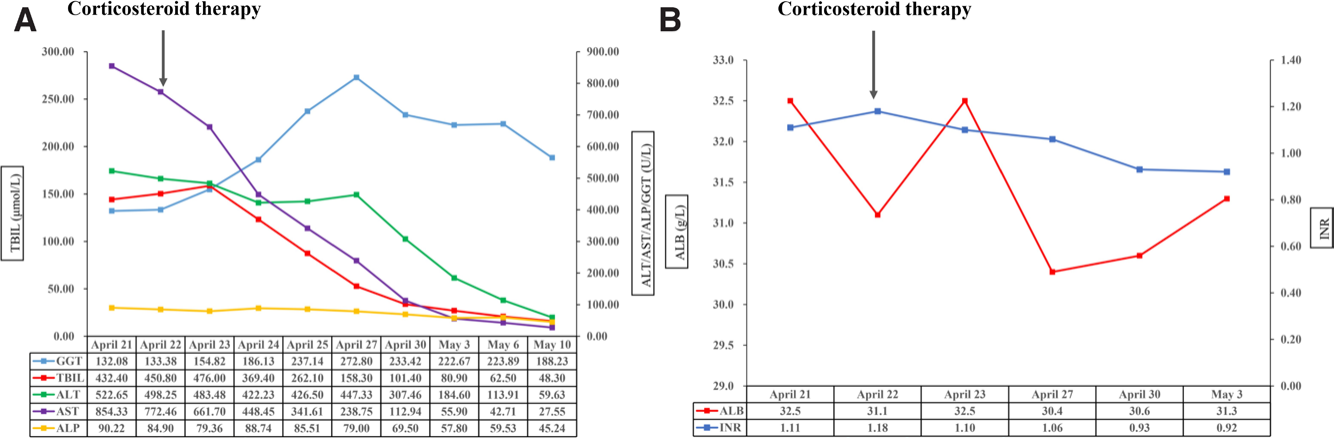
Changes in TBIL, ALT, AST, ALP, GGT (A), ALB, and INR (B) during hospitalization. ALB = serum albumin, ALP = alkaline phosphatase, ALT = alanine aminotransferase, AST = aspartate transaminase, GGT = gamma glutamyl transferase, INR = international normalized ratio, TBIL = total bilirubin.

**Figure 2.**
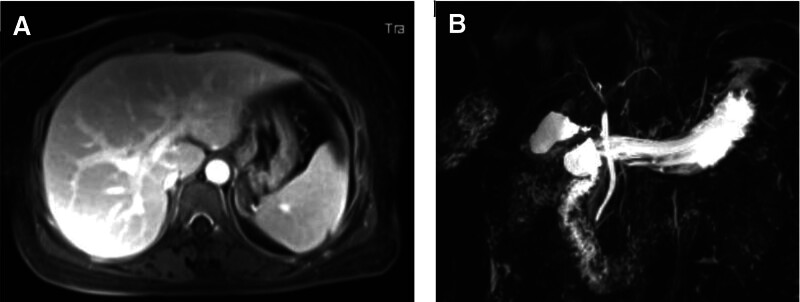
Magnetic resonance imaging (A) and magnetic resonance cholangiography (B).

A 55-year-old female patient developed progressive jaundice on March 2021 and underwent laboratory examinations at the local hospital showing that total bilirubin (TBIL) was 134.40 μmol/L (reference range: 2–20 μmol/L), alanine aminotransferase (ALT) was 1492 U/L (reference range: 0–40 U/L), aspartate transaminase (AST) was 1125 U/L (reference range: 0–40 U/L), alkaline phosphatase (ALP) was 125.88 U/L (reference range: 40–150 U/L), gamma glutamyl transferase (GGT) was 307 U/L (reference range: 0–50 U/L), and viral markers for hepatitis A (IgM anti-HAV), hepatitis E (IgM anti-HEV), hepatitis B (HBsAg), and hepatitis C (anti-HCV) were negative. Her clinical course did not improve after symptomatic treatment. On April 19, 2021, the patient was transferred to our department. She did not have any history of alcohol abuse, underlying diseases, or liver diseases. She denied the use of drugs and herbal medicines, but took Mega Defends X to enhance immunity during the period from January 2021 to March 2021 before the occurrence of jaundice. On physical examination, vital signs were as follows: blood pressure, 111/85 mm Hg; heart rate, 110 beats per minute; respiratory rate, 20 beats per minute; temperature was 37.1 °C. Scleral icterus was observed. There was no skin rash, abdominal tenderness, hepatomegaly, or splenomegaly. Her body mass index was 21 kg/m^2^. Serologies for Epstein–Barr virus, cytomegalovirus, and antinuclear, anti-liver kidney microsome, anti-smooth muscle, and anti-mitochondrial antibodies were negative. Laboratory examinations at her admission showed that eosinophil count was 0.09 × 10^9^/L (reference range: 0.02–0.52 × 10^9^/L), white blood cell was 8.1 × 10^9^/L (reference range: 3.9–9.5 × 10^9^/L), TBIL was 432.40 μmol/L (reference range: 5.1–22.2 μmol/L), ALT was 522.65 U/L (reference range: 7–40 U/L), AST was 854.33 U/L (reference range: 13–35U/L), ALP was 90.22 U/L (reference range: 35–135 U/L), GGT was 132.08 U/L (reference range: 7–45 U/L), serum albumin (ALB) was 32.5 g/L (reference range: 40–55 g/L), and international normalized ratio (INR) was 1.11 (Fig. 1). Magnetic resonance imaging showed a normal size and shape of the liver, and magnetic resonance cholangiography revealed no obstruction of the bile duct (Fig. 2). The patient and her family refused liver biopsy. The updated Roussel Uclaf Causality Assessment Method (RUCAM) score was 9,^[7]^ consistent with highly probable causality grading (Table 1). Thus, a diagnosis of HILI was considered with an R score of 19.55, which is consistent with hepatocellular pattern. Initially, intravenous infusion of ademetionine 1,4-butanedisulfonate, polyene phosphatidylcholine, and glutathione were given.

**Table 1:** Detailed RUCAM score for our patient*.

Items for hepatocellular injury	Score	Result
1 Time to onset from the beginning of the drug/herb		+2
5–90 days (rechallenge: 1–15 days)	+2	√
<5 or > 90 days (rechallenge: >15 days)	+1	
≤15 days (except for slowly metabolized chemicals: >15 days)	+1	
2 Course of ALT after cessation of the drug/herb		+3
Decrease ≥ 50% within 8 days	+3	√
Decrease < 50% within 30 days	+2	
No information or continued drug use	+0	
Decrease ≥ 50% after the 30th day	+0	
Decrease < 50% after the 30th day or recurrent increase	−2	
3 Risk factors		+1
Alcohol use (current drinks/d: >2 for women, >3 for men)†	+1	
Alcohol use (current drinks/d: ≤2 for women, ≤3 for men)†	+0	
Age ≥ 55 years	+1	√
Age < 55 years	+0	
4 Concomitant drug(s)/herb(s)		+0
None or no information	+0	√
Concomitant drug/herb with incompatible time to onset	+0	
Concomitant drug/herb with compatible or suggestive time to onset	−1	
Concomitant drug/herb known as hepatotoxin and with compatible or suggestive time to onset	−2	
Concomitant drug/herb with evidence for its role in this case (positive rechallenge or validated test)	−3	
5 Search for alternative causes		+2
	Negative	Positive
Group I (7 causes)		
HAV: Anti-HAV-IgM	√	
HBV: HBsAg, anti-HBc-IgM, HBV-DNA	√	
HCV: Anti-HCV, HCV-RNA	√	
HEV: Anti-HEV-IgM, anti-HEV-IgG, HEV-RNA	√	
Hepatobiliary sonography/color Doppler sonography of liver vessels/endosonography/CT/MRC	√	
Alcoholism (AST/ ALT ≥ 2)	√	
Acute recent hypotension history (particularly if underlying heart disease)	√	
Group II (5 causes)		
Complications of underlying disease(s) such as sepsis, metastatic malignancy, autoimmune hepatitis, chronic hepatitis B or C, primary biliary cholangitis or sclerosing cholangitis, genetic liver diseases	√	
Infection suggested by PCR and titer change for		
CMV (anti-CMV-IgM, anti-CMV-IgG)	√	
EBV (anti-EBV-IgM, anti-EBV-IgG)	√	
HSV (anti-HSV-IgM, anti-HSV-IgG)	√	
VZV (anti-VZV-IgM, anti-VZV-IgG)	√	
Evaluation of group I and II		
All causes-groups I and II: reasonably ruled out	+2	√
The 7 causes of group I ruled out	+1	
6 or 5 causes of group I ruled out	+0	
<5 causes of group I ruled out	−2	
Alternative cause highly probable	−3	
6 Previous hepatotoxicity of the drug/herb		+1
Reaction labeled in the product characteristics	+2	
Reaction published but unlabeled	+1	√
Reaction unknown	+0	
7 Response to unintentional re-exposure		+0
Doubling of ALP with the drug/herb alone, provided ALP below 2N before re-exposure	+3	
Doubling of ALP with the drugs(s)/herbs(s) already given at the time of first reaction	+1	
Increase of ALP but less than N in the same conditions as for the first administration	−2	
Other situations	+0	√
Total score for the case		9

ALT = alanine aminotransferase; AST = aspartate aminotransferase: CMV = cytomegalovirus; CT = computer tomography; DILI = drug-induced liver injury; EBV = Epstein–Barr virus; HAV = hepatitis A virus; HBc = hepatitis B core; HBsAg = hepatitis B antigen; HBV = hepatitis B virus; HCV = hepatitis C virus; HEV = hepatitis E virus; HILI = herb-induced liver injury; HSV = Herpes simplex virus; MRC = magnetic resonance cholangiography; N = upper limit of the normal range; RUCAM = Roussel Uclaf Causality Assessment Method; VZV = Varicella zoster virus.

* Adapted from an open access report on the updated RUCAM^[7]^.

† Each drink was 10 g ethanol.

On April 22, TBIL was 450.8 μmol/L, ALT was 498.25 U/L, AST was 772.46 U/L, ALP was 84.90 U/L, GGT was 133.38 U/L, ALB was 31.1 g/L, and INR was 1.18, indicating a deterioration of HILI (Fig. 1). At the same day, methylprednisolone was intravenously given at a dosage of 40 mg/d. On April 25, TBIL was 262.10 μmol/L, ALT was 426.50 U/L, AST was 341.61 U/L, AKP was 85.51 U/L, and GGT was 237.14 U/L (Fig. 1). On May 10, TBIL was 48.30 μmol/L, ALT was 59.63 U/L, AST was 27.55 U/L, ALP was 45.24 U/L, GGT was 188.23 U/L (Fig. 1). Then, she was discharged. The dosage of methylprednisolone was gradually reduced for 2 weeks and completely stopped 3 months after discharge. Liver tests remained normal during a 15-month follow-up period.

## 3. Discussion

Herbal products are often considered to be safe, but carry a potential risk of damage to the liver and other organs.^[8]^ In our case, other definite causes of liver injury were excluded based on clinical history, laboratory examinations, and imaging findings. After reviewing the instructions, Mega Defends X is an herbal product, consisting of vitamins C, B1, B2, B6, B12, and E, selenium, coenzyme Q10, baicalin, and catechin. Notably, baicalin and catechin may cause significant liver injury.^[9,10]^ However, Puri et al found that liver injury may not be related to baicalin, but other drugs concomitantly taken, after reviewing the cases of liver injury induced by baicalin.^[11]^ In addition, Puri et al also assessed the change of liver function in patients on long-term baicalin administration and suggested no evidence about hepatotoxicity of baicalin.^[11]^ Indeed, the United States Pharmacopeia deemed that the occurrence of severe hepatotoxicity might be associated with catechin.^[12]^ But hepatotoxicity may be idiosyncratic, but not dose-dependent.^[13]^ Besides, pesticide residues and bacterial contamination in the production process of herbal products may also cause liver injury.^[13]^ Finally, we suspected that the use of Mega Defends X is the highly probable source of the liver injury.

Currently, it is still challenging to make an accurate diagnosis of HILI. This might be due to the absence of specific biomarkers and manifestations for diagnosis of HILI. Its diagnosis is primarily based on the disease course after exclusion of other liver diseases.^[7]^ The challenge period from initial ingestion of suspected herb products to the onset of HILI can range from a few days to months, and HILI mostly occurs in the first 3 months of ingestion.^[6]^ As a well-known scale for diagnosis of HILI, RUCAM includes 7 different domains, and provides a framework for a more objective evaluation in suspected cases of HILI. The causality between suspected herbal products and liver injury is classified into highly probable (score ≥ 9), probable (6–8), possible (3–5), unlikely (1–2), and excluded (≤0) based on the updated RUCAM score.^[7]^ In our patient, the RUCAM score was 9, consistent with highly probable causality.

HILI can be classified into hepatocellular injury (*R* ≥ 5), cholestatic injury (*R* ≤ 2), and hepatocellular–cholestatic mixed injury (2 < *R* < 5).^[7]^ The *R* value is defined as actual ALT/upper limit of normal divided by actual ALP/ upper limit of normal. The actual ALT and ALP should be initial.^[14]^ In our patient, the initially actual ALT and ALP levels were 1492 U/L and 125.99 U/L, respectively. In our patient, the R score was 19.55, indicating hepatocellular HILI. Drugs should be selected according to the clinical pattern of HILI.^[15]^ For hepatocellular HILI, a priority should be given to hepatoprotective drugs, including N-acetylcysteine, glutathione, magnesium isoglycyrrhizinate, and polyene phosphatidylcholine. For cholestatic injury HILI, a priority should be given to anticholestatic drugs, including ursodeoxycholic acid and S-adenosylmethionine. Our patient with hepatocellular HILI who presented with jaundice was initially treated with ademetionine 1,4-butanedisulfonate, polyene phosphatidylcholine, and glutathione.

According to the Chinese practice guideline, the severity of HILI was classified into 5 grades, including mild (TBIL > 42.75 μmol/L with INR < 1.5), moderate (TBIL > 42.75 μmol/L with INR ≥ 1.5), and severe liver injury (TBIL > 85.5 μmol/L with/without INR ≥ 1.5), ALF (TBIL > 171 μmol/L with INR ≥ 2.0), and death.^[14]^ At our patient’s admission, TBIL level was 423.4 μmol/L, but INR was 1.11, which indicated severe liver injury. Silymarin, bicyclol, magnesium isoglycyrrhizinate, and N-acetylcysteine are often suitable for acute HILI.^[16]^ If TBIL level was not reduced after regular therapy, corticosteroids would be considered.^[17]^ However, the role of corticosteroid for HILI is controversial. Hou et al demonstrated that corticosteroid therapy group showed a higher rate of disease resolution and shorter recovery time,^[18]^ which was similar to studies conducted by Niu et al^[19]^ and Wang et al.^[20]^ On the contrary, Karkhanis et al^[21]^ found that corticosteroids did not improve overall survival of HILI patients. This might be primarily because nearly all included patients were diagnosed with ALF. The severity of HILI seems to influence the efficacy of corticosteroids. In our patient, methylprednisolone as intravenous infusion (40 mg/d) was given since prior treatment ademetionine 1,4-butanedisulfonate, polyene phosphatidylcholine, and glutathione was not effective.

A therapy with gradually tapering off the dosage of corticosteroid is recommended in clinical practice, because its abrupt withdrawal may result in the recurrence of the liver injury.^[17]^ In our patient, the dosage of methylprednisolone was gradually reduced and completely stopped 3 months after discharge. Corticosteroids may cause infection, gastrointestinal bleeding, diabetes mellitus, hypertension, psychosis, moon face, and osteoporosis.^[22]^ However, previous studies found that corticosteroids failed to increase the incidences of infection and gastrointestinal bleeding in patients with HILI.^[21,23]^ Diabetes mellitus, psychosis, and hypertension also disappeared after withdrawal of corticosteroid.^[24]^ Although the adverse events of corticosteroids are treatable, this should be kept in mind and take prompt interventions. Our patient did not develop any adverse events during treatment of the corticosteroid. The use of corticosteroid seems to be safe in our patient.

## 4. Conclusion

Our case report suggests the probability of developing HILI secondary to herbal products such as the traditional Chinese medicine, Mega Defends X. Therefore, herbal products should be taken cautiously. Corticosteroids are potentially effective and safe in patients with severe HILI. In future, randomized clinical trials are necessary to evaluate the efficacy and safety of corticosteroids for HILI. The updated RUCAM represents an important clinical framework for HILI diagnosis and helps physicians decide whether HILI is the most probable diagnosis.

## Acknowledgments

The authors would like to thank the patient involved in this study and our colleagues at the General Hospital of Northern Theater Command, Shenyang, China.

## Author contributions

**Conceptualization:** Xingshun Qi.

**Data curation:** Lu Chai, Ran Wang.

**Formal analysis:** Lu Chai.

**Software:** Ran Wang.

**Visualization:** Lu Chai.

**Writing – original draft:** Lu Chai, Xingshun Qi.

**Writing – review & editing:** Lu Chai, Ran Wang, Rolf Teschke, Shenghao Jin, Jiao Deng, Xingshun Qi.
